# Supplementary material for: SyNDI: synchronous network data integration framework
Source: BMC Bioinformatics. 2018 Nov 6;19:403. doi: 10.1186/s12859-018-2426-5 (PMC6219086; doi:10.1186/s12859-018-2426-5)
Supplement: Supplementary file 5 — Files of the example presented in Additional file 4. This additional file is a zip package that contains all files used in Additional file 4. Detailed descriptions of each of these files are presented in a README file included in this zip package. (GZ 30411 kb) [file 12859_2018_2426_MOESM5_ESM.gz › SyNDITools/Meme2Fimo/meme_4.9.0_4.tar.gz!/meme_4.9.0/doc/tomtom.html]

Tomtom - motif comparison tool


# The MEME Suite

## Motif-based sequence analysis tools

### Usage:

`tomtom [options] <query motifs>
<target motif database>+`

### Description

The Tomtom program searches one or more query motifs against one or
more databases of target motifs (and their DNA reverse complements), and
reports for each query a list of target motifs, ranked by *p*-value.
The *E*-value and the *q*-value of each match is also reported.
The *q*-value is the minimal false discovery rate at which the
observed similarity would be deemed significant. The output contains
results for each query, in the order that the queries appear in the input
file.

For a given pair of motifs, the program considers all offsets, while
requiring a minimum number of overlapping positions. For a given offset,
each overlapping position is scored using one of seven column similarity
functions defined below. Columns in the query motif that don't overlap the
target motif are assigned a score equal to the median score of the set of
random matches to that column. In order to compute the scores, Tomtom
needs to know the frequencies of the letters of the sequence alphabet in
the database being searched (the "background" letter frequencies). By
default, the background letter frequencies included in the MEME input
files are used. The scores of columns that overlap for a given offset are
summed. This summed score is then converted to a *p*-value. The
reported *p*-value is the minimal *p*-value over all possible
offsets. To compensate for multiple testing, each reported *p*-value
is converted to an *E*-value by multiplying it by twice the number of
target motifs. As a second type of multiple-testing correction,
*q*-values for each match are computed from the set of
*p*-values and reported.

### Inputs

#### Query Motifs

A file containing one or more motifs in MEME format. Each of these motifs will be searched against the
target databases. If you only wish to search with a subset of these
motifs then look into the -m and
-mi options.

#### Target Motif Databases

One or more files containing one or more motifs in
MEME format.

### Output

Tomtom writes its output to files in a directory named
`tomtom_out`, which it creates if necessary. (You can also
cause the output to be written to a different directory; see
-o and -oc, below.)

The main output file is named `tomtom.html` and can be viewed
with a web browser. The `tomtom.html` file is created from the
`tomtom.xml` file. An additional file, `tomtom.txt`,
contains a simplified, text-only version of the output. (See
`-text`, below, for the text output format.)

For each query-target match, two additional files containing LOGO
alignments may also be written -- an encapsulated postscript file
(`.eps`) if the -eps flag is
specified and a portable network graphic file (`.png`) if the
-png flag is specified. An install of
ghostscript is required to create the png file.

Only matches for which the significance is less than or equal to the
threshold set by the -thresh switch will be
shown. By default, significance is measured by *q*-value of the
match. The *q*-value is the estimated false discovery rate if the
occurrence is accepted as significant. See Storey JD, Tibshirani R,
"Statistical significance for genome-wide studies".
*Proc. Natl Acad. Sci. USA (2003) 100:9440–9445*

### Options

| Option | Parameter | Description | Default Behaviour |
| --- | --- | --- | --- |
| Input | | | |
| --- | --- | --- | --- |
| -bfile | background file | Load the background frequencies from the file named background file. | Background frequencies will be derived from the first target database. |
| -m | id | The name of a motif in the query file that will be used. This option may be repeated multiple times. | If both this option and the related -mi is unused then all motifs in the query file will be used. |
| -mi | index | The offset in the query file of a motif that will be used. This option may be repeated multple times. | If both this option and the related -m is unused then all motifs in the query file will be used. |
| -query-pseudo | count | This option adds the specified pseudocount to each count in the each query matrix. | No pseudocount is added to the query matricies. |
| -target-pseudo | count | This option adds the specified pseudocount to each count in the each query matrix. | No pseudocount is added to the target matrices. |
| Output | | | |
| --- | --- | --- | --- |
| -o | name | Create a folder called name and write output files in it. If the output folder already exists then the program will exit without writing anything. This option is not compatible with -oc as only one output folder is allowed. | The program behaves as if `-oc tomtom_out` had been specified. |
| -oc | name | Create a folder called name but if it already exists allow overwriting the contents. This option is not compatible with -o as only one output folder is allowed. | The program behaves as if `-oc tomtom_out` had been specified. |
| -png |  | Output motif logo alignment images in portable network graphics (png) format. This format is useful for display on websites. | Images are not output in png format. |
| -eps |  | Output motif logo alignment images in Encapsulated Postscript (eps) format. This format is useful for inclusion in publications as it is a vector graphics format and can be easily scaled. | Images are not output in eps format. |
| -text |  | This option causes Tomtom to print just a tab-delimited text file to standard output. The output begins with a header, indicated by leading "#" characters. This is followed by a single title line, and then the actual values. The columns are Column \| Contents; 1 \| Query motif name; 2 \| Target motif name; 3 \| Optimal offset: the offset between the query and the target motif; 4 \| *p*-value; 5 \| *E*-value; 6 \| *q*-value; 7 \| Overlap: the number of positions of overlap between the two motifs.; 8 \| Query consensus sequence.; 9 \| Target consensus sequence.; 10 \| Orientation: Orientation of target motif with respect to query motif. | The program runs as normal. |
| -no-ssc |  | This option causes the LOGOs in the LOGO alignments output by Tomtom not to be corrected for small-sample sizes. By default, the height of letters in the LOGOs are reduced when the number of samples on which a motif is based (`nsites` in the MEME motif) is small. The default setting can cause motifs based on very few sites to have "empty" LOGOs, so this switch can be used if your query or target motifs are based on few samples. | Small sample correction is used. |
| Scoring | | | |
| --- | --- | --- | --- |
| -incomplete-scores |  | Compute scores using only aligned columns. | Take into account columns that don't align. |
| -thresh | value | Only report matches with significance values ≤ value. Unless the `-evalue` option is specifed then this value must be smaller than or equal to 1. | A threshold of 0.5 is used. |
| -evalue |  | Use the *E*-value of the match as the significance threshold | Use the *q*-value as the significance threshold |
| -dist | allr\|ed\|kullback\|pearson\|sandelin | Code \| Name; allr \| Average log-likelihood ratio; ed \| Euclidian distance; kullback \| Kullback-Leibler divergence; pearson \| Pearson correlation coefficient; sandelin \| Sandelin-Wasserman function Detailed descriptions of these functions can be found in the published description of Tomtom. | Pearson correlation coefficient is used by default. |
| -internal |  | This parameter forces the shorter motif to be completely contained in the longer motif. | The shorter motif may extend outside the longer motif. |
| -min-overlap | min overlap | Only report motif matches that overlap by min overlap positions or more. In case a query motif is smaller than min overlap, then the motif's width is used as the minimum overlap for that query. | A minimum overlap of 1 is required. |
| Miscellaneous | | | |
| --- | --- | --- | --- |
| -verbosity | 1\|2\|3\|4\|5 | This option changes the level of detail of messages printed. At level 1 only critical errors are reported whereas at level 5 everything is printed. | The default is 2. |

Authors:
Shobhit Gupta (shobhitg@u.washington.edu),
Timothy Bailey (tbailey@imb.uq.edu.au),
Charles E. Grant (cegrant@gs.washington.edu) and
William Noble (noble@gs.washington.edu).
